# Vomiting from Hawking Mucus

**Published:** 1898-02

**Authors:** A. B. Eadie

**Affiliations:** Ithaca, N. Y.


### VOMITING FROM HAWKING MUCUS.

A. B. Eadie, Ithaca, N. Y.

A few years ago I was applied to by a patient who suffered from vomiting his breakfast, caused by attempting to clear his throat of an offensive catarrhal mucus. I made a number of prescriptions that did little but palliate, and finally for some intercurrent malady gave him Euphrasia 2c. He returned after a few days to say "that medicine cured him of vomiting his breakfast." This fall I had a patient who complained that every morning on his way to work he vomited his breakfast on clearing his throat of an offensive phlegm. He suffered all last winter from the same trouble, and now it had come on again. Euphrasia 2c. removed the trouble at once, and he has been free for about a month.

